# A MRI-Compatible Combined Mechanical Loading and MR Elastography Setup to Study Deformation-Induced Skeletal Muscle Damage in Rats

**DOI:** 10.1371/journal.pone.0169864

**Published:** 2017-01-11

**Authors:** Jules L. Nelissen, Larry de Graaf, Willeke A. Traa, Tom J. L. Schreurs, Kevin M. Moerman, Aart J. Nederveen, Ralph Sinkus, Cees W. J. Oomens, Klaas Nicolay, Gustav J. Strijkers

**Affiliations:** 1 Biomedical NMR, Biomedical Engineering, Eindhoven University of Technology, Eindhoven, The Netherlands; 2 Biomedical Engineering and Physics, Academic Medical Center, Amsterdam, The Netherlands; 3 Soft Tissue Biomechanics and Engineering, Biomedical Engineering, Eindhoven University of Technology, Eindhoven, The Netherlands; 4 Center for Extreme Bionics, Media lab, MIT, Cambridge, MA, United States of America; 5 Department of Radiology, Academic Medical Center, Amsterdam, The Netherlands; 6 Image Sciences & Biomedical Engineering, King’s College London, London, United Kingdom; Universite de Technologie de Compiegne, FRANCE

## Abstract

Deformation of skeletal muscle in the proximity of bony structures may lead to deep tissue injury category of pressure ulcers. Changes in mechanical properties have been proposed as a risk factor in the development of deep tissue injury and may be useful as a diagnostic tool for early detection. MRE allows for the estimation of mechanical properties of soft tissue through analysis of shear wave data. The shear waves originate from vibrations induced by an external actuator placed on the tissue surface. In this study a combined Magnetic Resonance (MR) compatible indentation and MR Elastography (MRE) setup is presented to study mechanical properties associated with deep tissue injury in rats. The proposed setup allows for MRE investigations combined with damage-inducing large strain indentation of the Tibialis Anterior muscle in the rat hind leg inside a small animal MR scanner. An alginate cast allowed proper fixation of the animal leg with anatomical perfect fit, provided boundary condition information for FEA and provided good susceptibility matching. MR Elastography data could be recorded for the Tibialis Anterior muscle prior to, during, and after indentation. A decaying shear wave with an average amplitude of approximately 2 μm propagated in the whole muscle. MRE elastograms representing local tissue shear storage modulus G_d_ showed significant increased mean values due to damage-inducing indentation (from 4.2 ± 0.1 kPa before to 5.1 ± 0.6 kPa after, p<0.05). The proposed setup enables controlled deformation under MRI-guidance, monitoring of the wound development by MRI, and quantification of tissue mechanical properties by MRE. We expect that improved knowledge of changes in soft tissue mechanical properties due to deep tissue injury, will provide new insights in the etiology of deep tissue injuries, skeletal muscle damage and other related muscle pathologies.

## Introduction

Sustained mechanical loading and deformations of skeletal muscle in the proximity of bony structures may lead to damage in the deep soft tissue layers. This category of injury is recognized as a special type of pressure ulcer by the National and European Pressure Ulcer Advisory Panel (NPUAP/EPUAP) and is commonly referred to as deep tissue injury [1–5]. The pressure ulcer may initially stay invisible and remain undetected for days, until the injury becomes visible as a purple or maroon discolored skin spot, thereafter rapidly developing into a severe and difficult to heal Stage III or Stage IV pressure ulcer [6,7]. The populations at high risk for developing deep tissue injury include intensive- and acute-care patients, hospice patients, as well as people that require long-term care, for example after a spinal cord injury or stroke [8]. Common risk areas are the coccyx, sacrum, buttocks, knee, and heel [9,10]. Deep tissue injury is related to increased morbidity and mortality [7,11–13]. Cost-of-illness studies for pressure ulcers report a significant cost burden for society. In particular for deep tissue injuries; with wound closing times between 127 up to 155 days, typical treatment costs are 10,000–15,000 GBP per ulcer [14–16].

Clinicians and experienced wound nurses have noticed differences in mechanical stiffness between healthy and injured muscle in early stages of deep tissue injury development by means of palpation [17,18]. Finite element analysis (FEA) studies have shown that changes in mechanical properties of the affected tissue may lead to larger deformations and aggravated wounds [19,20]. Thus, the changes in mechanical properties of soft tissues have been proposed as one of the risk factors in the development of deep tissue injury, and analysis of tissue stiffness may be useful as a diagnostic tool for early detection [21]. However, the spatial-temporal changes in muscle mechanical properties from deformation-induced injury have never been systematically studied and quantified in a controlled setting.

A rat model for deep tissue injury has been studied extensively before, using MRI and FEA methods [22–32]. This well-established animal model of deep tissue injury involves the compression of the rat Tibialis Anterior (TA) muscle with a custom loading device that can be placed in an MRI scanner for imaging during the development of the wound [24]. Maps of the global transversal relaxation time T_2_ were used as a readout of muscle edema as a consequence of damage and inflammation after load release[23,26,31,33]. T_2_ is generally accepted as quantitative damage marker in musculoskeletal MRI [34–39]. Animal-specific FEA models, where the geometry and loading were derived from MRI, supplied estimations of the local tissue deformations. This combined experimental-numerical approach resulted in new understandings of the mechanical boundary conditions that contribute to the development of deep tissue injury [32,40].

The animal model raised new questions about the role of the muscle tissue mechanical properties in the etiology of wound development. Particularly, it is currently not known to which extent muscle stiffness alters under compression during the development of the injury and in the early healing stage, nor how stiffness changes contribute to the development of severe wounds. Since stiffness changes have been reported during tissue injury development, assessment of changes in tissue stiffness could serve a role in diagnosis of deep tissue injury [7,19,41–44].

Magnetic Resonance Elastography (MRE) is an MRI technique for measuring mechanical properties of soft tissue by measuring mechanically induced shear waves [45,46]. The small strain harmonic shear waves, induced by an external MRI-compatible actuator, are imaged using a motion-encoded phase contrast MRI sequence. By inversion of the wave image data, Hookean viscoelastic mechanical properties, such as the shear storage modulus G_d_, can be quantified [47,48]. In liver diseases MRE proves to be a technique of great diagnostic and etiologic value [49–52], and is also increasingly utilized for use in the kidney, spleen, brain, breast, heart, lungs, prostate, and skeletal muscle [53–61]. MRE studies of skeletal muscle have primarily focused on healthy skeletal muscle to better understand the complex anisotropic viscoelastic mechanical properties and to assess changes in muscle shear stiffness during active and passive contraction [62–70]. In a number of studies MRE has been applied to characterize muscle disease and has proven to have added value in addition to the more conventional MRI methods; MRE is proposed as objective evaluation method to monitor the efficacy of treatments for pathologic or injured muscle [71–78].

The application of MRE to assess deep tissue injury was proposed in several papers, but it has, to the best of our knowledge not been performed yet [19,20,41–43,79]. In this paper, we present an MRI-compatible setup for studying the mechanical properties of rat TA muscle during the development of deep tissue injury. The setup allows for controlled deformation under MRI-guidance, monitoring of the wound development by MRI, and quantification of tissue mechanical properties and potential changes therein by the MRE technique.

## Methods

### Setup

**Fig 1:**
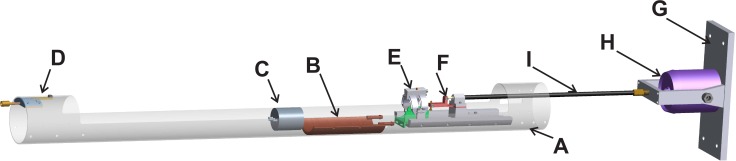
Overview of the MR compatible indentation and MRE setup. Following parts are labeled: Fiberglass tube (**A**), 3D-printed heating blanket (**B**), anesthesia mask (**C**), fixation block (**D**), indentor (**E**), MRE actuator (**F**), shaker base plate (**G**), electromagnetic shaker (**H**). Part **E** and **F** are shown in more detail in Fig 2.

A schematic illustration of the setup is shown in Fig 1. The setup is fixed in (**A**) a fiberglass tube with an outer diameter of 86 mm and a wall thickness of 2 mm. Inside the tube the following parts are mounted: (**B**) a 3D-printed water-circulated heating blanket for maintaining the rat body temperature, (**C**) an anesthesia mask with supply and exhaust of anesthesia gas for sedating the rat, (**D**) a fixation block to secure the setup in the MRI magnet, (**E**) the indentor, and (**F**) the MRE actuator. (**G**) A base plate with (**H**) electromagnetic shaker (LDS V201, Brüel and Kjaer, Royston, UK) is fixed to the wall of the Faraday cage at a safe distance from the stray field of the 7.0 T MRI magnet. The MRE actuator is brought into motion via (**I**) a splined transmission rod (type S518, Sullivan Products, Baltimore, US). The shaker is powered with an amplifier (QUAD 50E, Huntington, UK) and waveform generator (Agilent 33220A, Santa Clara, US) placed outside the Faraday cage. The shaker is air cooled with constant airflow. Synchronization of the MRE sequence with the shaker motion is performed via transistor-transistor logic (TTL) triggering. The TTL trigger signal is a 3V voltage level signal which is pulled to ground in case of a trigger. Signals from TTL triggering and waveform generator were visualized on an oscilloscope (Agilent InfiniVision 2000X oscilloscope, Santa Clara, US) to verify correct synchronization. The MRE actuator is roughly based on the design of Sinkus *et al*. that was used for MRE of colon tumors in mice and for studying an mdx mouse model of muscular dystrophy [71,80,81]. A photograph of a rat positioned in the setup can be found in the online supplement (S1 Fig).

**Fig 2:**
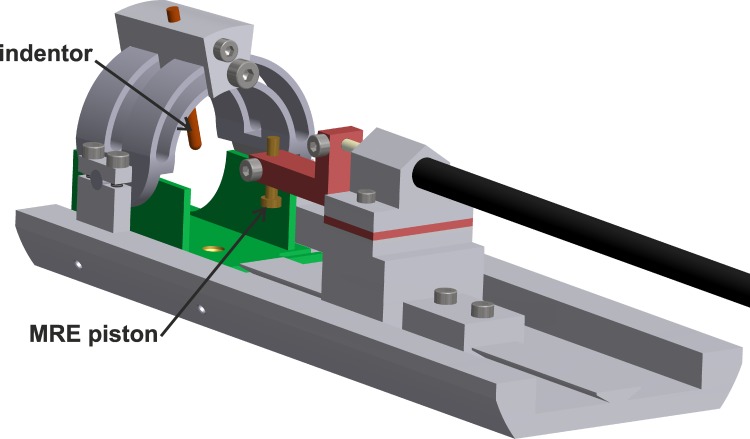
Detail of indentation and MRE actuator part of setup. Indentor and MRE piston are indicated with a label. Indentor can be positioned on the rats TA using the movable indentor holder. The MRE piston is coupled to the tendon at the distal side of the TA muscle. Overview figure indicating all parts is shown in online supplemental S2 Fig.

The indentation and MRE components (**E** and **F**) are shown in greater magnification and detail in Fig 2. The rat can be conveniently positioned in the setup using a right-left direction adjustable u-shaped profile with a cutout for the rat’s groin, which enables positioning of the right leg of the rat under the indentor (Fig 2 **indentor**). The indentor can be positioned on the rat TA muscle using a movable indentor holder and rotatable half arch. The indentor is a cylindrical rod (3 mm in diameter, 36.5 mm long) with a rounded head and is composed of two, screwed-together parts. The indentor assembly contains a hollow compartment, which is filled with an aqueous solution of 1 g/L CuSO_4_ for MRI visualization, inside a rigid solid rod. The MRE actuator is mounted on a dovetail profile allowing for adjustment in the axial direction. The height and depth of the indentation can be controlled by removing spacer plates or by altering the position of the MRE piston (Fig 2 **MRE piston**). If desired the MRE piston can be replaced by pistons of a different shape or size. The MRE piston is brought into motion via the drive rod attached to the electromagnetic shaker (Fig 1**H**) and cantilever. Detailed overview of all above described parts of the indentation and MRE components can be found in the online supplement (S2 Fig).

Indentation of the rat TA muscle is achieved by positioning the indentor above the TA muscle using the rotatable half-arch, by manually pushing the rod into the muscle, and by fixating it at the desired indentation depth and orientation. The MRE piston is softly coupled to the tendon at the distal side of the TA muscle. All parts are made of PET-P (polyethylene terephthalate polyester), or from PEEK (polyether ether ketone). Both materials are MRI-compatible, and have high mechanical strength, stiffness and creep resistance.

### Animal model

To investigate the feasibility of MRE based shear stiffness changes occurring during indentation induced damage 20 female Sprague-Dawley (SD) rats (11 to 13-week-old, ± 250 g, Charles River, Paris, France) were used. To optimize the settings of the experimental setup and the MR sequences 13 rats were used (results not shown). MRE measurements pre and post 2 h indentation was performed in 7 rats. In 1 of these rats MRE measurements were also performed during indentation. A duration of 2 h indentation was chosen based on previous studies, in which different durations of deformation, ischemia and reperfusion were studied [40]. The results of these experiments showed that deformation, ischemia and reperfusion all contribute to the damage process but that deformation induced damage is dominant for periods less than 2 h. Animals were housed under standard laboratory conditions with a 12 h light/dark cycle and were maintained on a standard diet and with access to water *ad libitum*. Rats were anesthetized with isoflurane (4.0 vol% for induction, 1.0–2.0 vol% for maintenance) in 0.6 L/min medical air. Buprenorphine (0.05 mg/kg s.c.) was administered for analgesia. Eye ointment was applied to prevent eye dehydration. The rat was placed in supine position in the MR-compatible setup. Body temperature was maintained at 35–37°C with the heating blanket and monitored with a rectal temperature sensor. Respiration was monitored with a balloon pressure sensor placed on the abdomen and maintained in a physiological range by adjusting the anesthesia. The right leg of the rat was shaved and positioned in the u-shaped profile filled with alginate molding substance for firm fixation and susceptibility matching. To assess the TA muscle during indentation the top of the alginate cast was removed making the TA muscle accessible to the indentor. After indentation the alginate cast was closed again. Indentation of the TA muscle for 2 h took place inside the MRI scanner. The MRE actuator was coupled to the tendon at the distal side of the TA muscle. After the measurements the animals were sacrificed by means of exsanguination from the inferior vena cava. This procedure was performed under anesthesia and after administration of analgesia. All animal experiments were approved by the Animal Care and Use Committee of Maastricht University, Maastricht, The Netherlands (protocol 2013–047, Maastricht University, Maastricht, The Netherlands) and performed in accordance with the Directive 2010/63/EU for animal experiments of the European Union.

### MRI

Measurements were performed with a 7.0 T small animal MRI scanner (Bruker BioSpin MRI GmbH, Ettlingen, Germany) equipped with a 660 mT/m, 4570 T/m/s gradient coil (BGA-12S HP, Bruker BioSpin MRI GmbH, Ettlingen, Germany). A 86-mm-inner-diameter quadrature transmit coil was used in combination with a 20-mm-diameter surface receive coil (Bruker BioSpin MRI GmbH, Ettlingen, Germany) placed on top of the TA muscle inside the indentation part of the setup. Anatomical images were acquired with T_1_-weighted MRI (scan parameters: sequence = FLASH 3D, field of view (FOV) = 6 x 6 x 6 cm^3^, acquisition matrix (MTX) = 192 x 192 x 192, echo time (TE) = 3.72 ms, repetition time (TR) = 16.84 ms, and acquisition time = 2:35 min). Alginate cast visualization was done with ultra-short echo time MRI (scan parameters: sequence = ultra-short echo time 3D, FOV = 6 x 6 x 6 cm^3^, MTX = 256 x 256 x 256, TE = 21 μs, TR = 2.69 ms, radial undersampling = 3 and acquisition time = 3:04 min) [82]. Elastography images were acquired with an in-house developed echo-planar-imaging (EPI) MRE sequence (sequence = SE-EPI-MRE 2D with fat suppression, number of slices = 18 slices, slice thickness = 1 mm, FOV = 3 x 6 cm^2^, MTX = 96 x 192, TE = 26.2 ms, TR = 1000 ms, actuator and motion encoding gradient (MEG) frequency = 900 Hz, number of EPI segments = 4, number of MRE offsets = 16, number of encoding directions = 3 (slice, phase, frequency encoding) plus reference, and acquisition time = 17:04 min). MRE during indentation was performed with a spin-echo based MRE sequence (sequence = SE-MRE 2D, number of slices = 10, slice thickness = 1 mm, FOV = 2.5 x 2.5 cm^2^, MTX = 128 x 128, TE = 21.1 ms, TR = 1000 ms, actuator and MEG frequency = 900 Hz, number of MRE offsets = 8, number of encoding directions = 1 (slice direction), and acquisition time = 17:05 min). Skeletal muscle edema was assessed with T_2_ mapping in both axial and coronal orientations (axial: sequence = MSME 2D with fat suppression, number of slices = 20, slice thickness = 1 mm, FOV = 2.5 x 2.5 cm^2^, MTX = 256 x 256, TE = 6.9–180.7 ms, number of echoes = 26, TR = 4000 ms, and acquisition time = 12:48 min; coronal: sequence = MSME 2D with fat suppression, number of slices = 18, FOV = 6 x 3 cm^2^, MTX = 512 x 256, TE = 10.2–203.5 ms, number of echoes = 20, TR = 3994 ms, and acquisition time = 12:54 min). All measurements were performed up to 90 min after the end of 2 h indentation.

### Data analysis

The MRE acquisitions provide phase data for the harmonic vibrations which are proportional to the harmonic displacements. By using an inversion algorithm the displacement data can be converted to elastograms, i.e. images representing the local tissue linear elastic shear storage modulus G_d_ (real part of the complex shear modulus G*). The inversion process assumes linear (visco-) elasticity, isotropy, and local homogeneity for all tissues, and aims to solve the following partial differential equation:

$$-\rho \omega^{2}q(x)=G^{*}\nabla^{2}q(x)$$ 1

Where G* is the complex shear modulus, *ρ* is the density, **q** is the curl of the complex displacement vector (***q***(*x*) = ∇ × *u*(*x*)) derived from the MRE phase data, and *ω* is the known angular frequency. In case of a continuous monochromatic wave the displacement is defined as:

$$u(x)=Ae^{ik∙x}$$ 2

with A the amplitude, and *k* the complex wave number. Combination of Eqs 1 and 2 produces:

$$G^{*}(\omega )=\frac{\rho \omega^{2}}{k^{2}}$$ 3

Combining Eq 3 with the fact that *k* = *β* + *iα*, the complex shear modulus G* can be split in terms of shear storage modulus G_d_ and shear loss modulus G_i_. Full details of the inversion algorithm implemented in the ROOT data analysis framework (ROOT 5.34/17, CERN, Meyrin, Switzerland) were previously described by Sinkus *et al* [83–85].

Quantitative T_2_ maps were obtained by pixel-wise fitting of the MR signal to $S=S_{0}e^{-TE/T_{2}}$ (Mathematica 10, Wolfram Research, Champaign, USA). Pixels with goodness of fit R^2^ < 0.7 and SNR < 4 were excluded from further analysis.

Region-of-interest (ROI) based analysis of the elastograms and T_2_ maps of the 6 rats measured before and after 2 h of indention was performed (Matlab R2016a, The Mathworks, Inc., Natick, Massachusetts, USA). An ROI was defined by manually outlining the TA muscle in the center slice. Mean values of G_d_, displacement amplitude A_tot_ and T_2_ were determined in this ROI before and after indentation. The percentages of significantly elevated G_d_ and T_2_ pixels were calculated by thresholding of pixels with values increased by 2 standard deviations as compared to the mean values pre-indentation. Mean, standard deviation (sd), and coefficient of variation (CV) were calculated to quantify the group average and variation at baseline, during and after indentation. Mean values of G_d_ and T_2_ were also determined in a circular ROI of 4 x the diameter of the indentor, positioned at the center of indentation. Paired sampled t-tests were conducted to test for significant differences between before and after indentation (Origin 2015, OriginLab Corporation, Northampton, USA).

## Results

The experimental methods presented here combine soft tissue indentation with MRE to assess the skeletal muscle shear storage modulus G_d_ before, during and after deep tissue injury development. Both the indentation device as well as the MRE actuator are designed to allow flexible positioning on top of the TA muscle in the hind leg of a rat and can be used before, during, and after indentation inside a small animal MRI scanner. The combination of an RF surface receiver coil and volume transmission coil resulted in high-quality MRI and MRE images of the whole TA muscle before, during, and after 2 h indentation. Fixation with alginate assures a good leg fixation with a perfect anatomical fit. In addition, it improves susceptibility matching enabling fast imaging with EPI-based sequences.

### MRI

**Fig 3:**
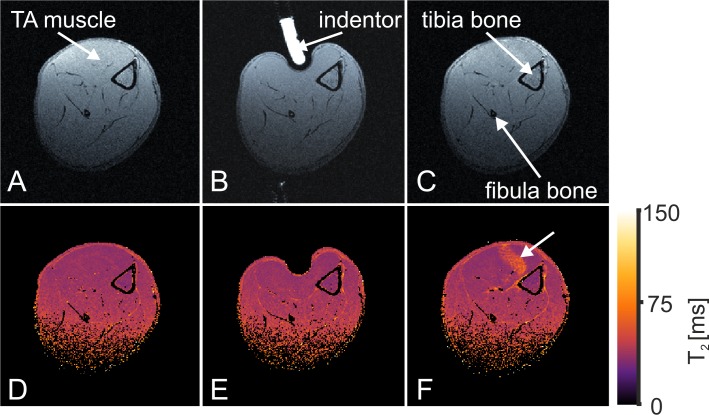
Anatomical images and T_2_ maps. Axial anatomical images (**A-C**) and T_2_ maps (**D-F**) of the hind limb before, during, and after indentation, respectively.

The manually set indentation depth can accurately be determined since the indentor is visible in the MR images (Fig 3B). Indentation depth was varied between rats resulting in different degrees of deep tissue injury in the TA muscles. Fig 3 shows representative axial anatomical T_1_-weighted images of a rat leg (A) pre, (B) during, and (C) post indentation together with the corresponding quantitative (D-F) T_2_ maps of a slice through the center of the region of deformation. The indentor pressing in the TA muscle is clearly visible in the T_1_-weighted images (arrow). The tibia bone, fibula bone, and skin contour provide anatomical landmarks from which indentation depth can be estimated. Compression of the TA muscle between the indentor and tibia bone is seen in (B). Elevated T_2_ values compared to pre indentation are seen in the T_2_ map post indentation (Fig 3F). These increased T_2_ values are indicative for the formation of edema and skeletal muscle damage as validated before [25,26,40].

**Fig 4:**
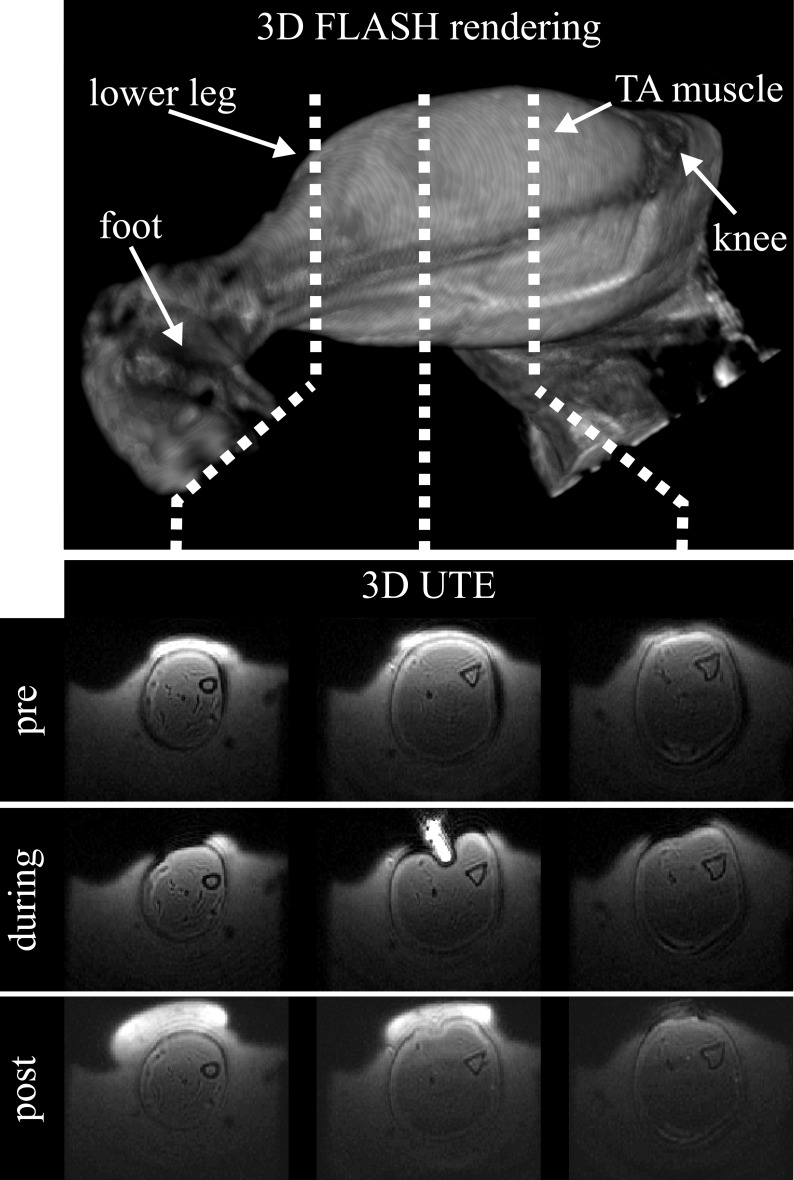
Alginate cast visualization. Axial 3D ultra-short echo time images of the alginate cast surrounding the rat leg, pre, during and post indentation. FLASH 3D rendering shows anatomical landmarks and location of selected 3D ultra-short echo time images.

Several axial slices of the 3D ultra-short echo time images of the alginate cast around the leg before, during, and after indentation are shown in Fig 4. Due to its short T_2_ relaxation time, the alginate is only visible on the ultra-short echo time scans and not on the regular T_1_-weighted and T_2_-weighted scans. The presence of alginate therefore does not interfere with the anatomical scans and the MRE measurements, and improves image quality by providing good susceptibility matching between the leg and its surroundings. During indentation (Fig 4 during) the top of the alginate cast was removed. Newly applied alginate is brighter on the post-indention ultra-short echo time scan (Fig 4 post). The cast closely follows the anatomy of the leg and little displacement of the leg between the three conditions is seen. The contours and the rigid fixation by the cast provide essential boundary conditions for FEA calculations of deformation related strains, as previously demonstrated [86].

### Magnetic resonance elastography

**Fig 5:**
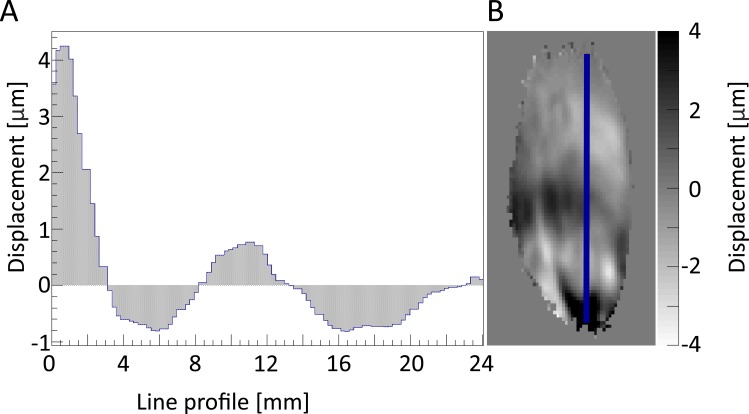
Displacement field of z-direction with line profile. (**A**) Shows the displacement in μm along the line profile illustrated as blue line in the (**B**) displacement field image of the z direction in the TA muscle.

Fig 5A displays a representative displacement wave in non-damaged TA muscle along the blue line in the displacement field of Fig 5B. A sinusoidal displacement wave with decaying amplitude was observed along the entire path of the travelling wave. Mean (A_tot_) and maximum (A_max_) TA displacement amplitudes for all 6 animals measured before and at all time points after indentation are listed in Table 1. The displacement amplitude was in the range of 0.5–10 μm (mean 2 μm).

**Table 1:** Mean and maximum displacement amplitude A_tot_ before, 30, 60 and 90 min after end of indentation per animal and mean ± sd of the group.

animal	before		30 min after		60 min after		90 min after	
	A_tot_	A_max_	A_tot_	A_max_	A_tot_	A_max_	A_tot_	A_max_
	[μm]	[μm]	[μm]	[μm]	[μm]	[μm]	[μm]	[μm]
**1**	1.5	7.2	1.6	4.2	1.9	5.1		
**2**	3.7	9.9	2.8	8.0	2.3	5.2		
**3**	1.1	5.1	1.3	3.9	1.5	3.9	1.6	3.2
**4**	2.2	6.9	1.8	6.9	1.7	7.9	1.8	8.4
**5**	2.5	8.9	2.8	7.3	2.7	9.7	3.1	7.8
**6**	1.3	5.8	1.8	5.3	1.8	4.2	1.6	3.9
**mean**	2.0	7.3	2.0	5.9	2.0	6.0	2.0	5.8
**sd**	1.0	1.8	0.6	1.7	0.5	2.3	0.7	2.7

**Fig 6:**
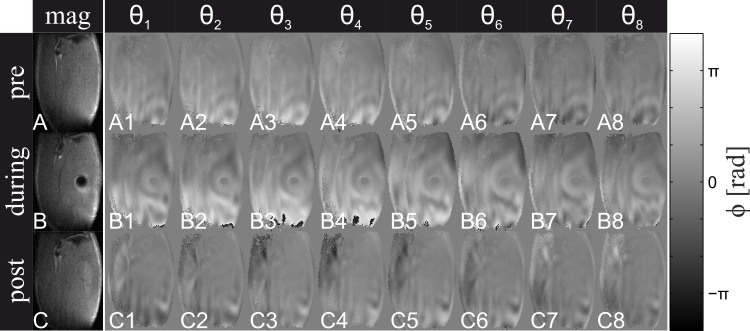
MRE pre, during post indentation. SE-MRE magnitude (mag) and wave (θ_1_—θ_8_) images before, during and after indentation. Wave images θ_1_, θ_2_, θ_3_, θ_4_, θ_5_, θ_6_, θ_7_, and θ_8_ correspond to 0^0^, 45^0^, 90^0^, 135^0^, 180^0^, 225^0^, 270^0^, 315^0^ wave phase, respectively.

In Fig 6 eight offsets of coronal SE-MRE magnitude images (mag) and wave images (θ_1_—θ_8_) are shown before, during, and after indentation. Distinct wave propagation was observed during the equally distributed time offsets θ_1_—θ_8_ of the 900 Hz wave with a time resolution of 0.1389 ms. In Fig 6B the dark spot in the TA corresponds to the location of the indentor. The wave pattern in (Fig 6B1–6B8) during indentation was distinctly different compared to pre indentation (Fig 6A1–6A8). The changes in the wave patterns relate to changes in tissue elasticity and geometry due to indentation, and also by reflection of shear waves at the indentor surface. Post indentation (Fig 6C1–6C8) the waves show a different pattern compared to pre indentation (Fig 6A1–6A8).

**Fig 7:**
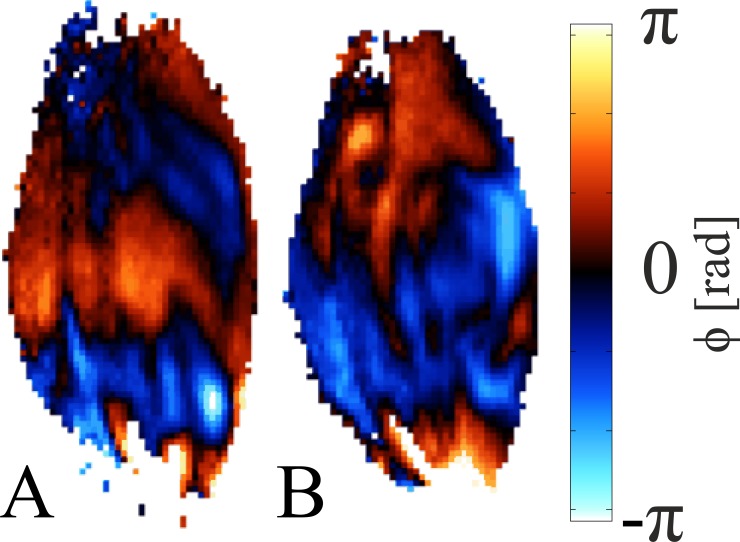
MRE pre and post indentation. Snapshot of a 16 offset 900 Hz SE-EPI-MRE (**A**) before and (**B**) after indentation.

Fig 7 shows one offset of a 16 offsets SE-EPI-MRE data set acquired (A) before and (B) after 2 h indentation, respectively. A movie of all offsets is added in the online supplemental of this article (S2 Fig). A distinct change in the wave pattern was observed due to altered tissue stiffness of the wound caused by indentation.

**Fig 8:**
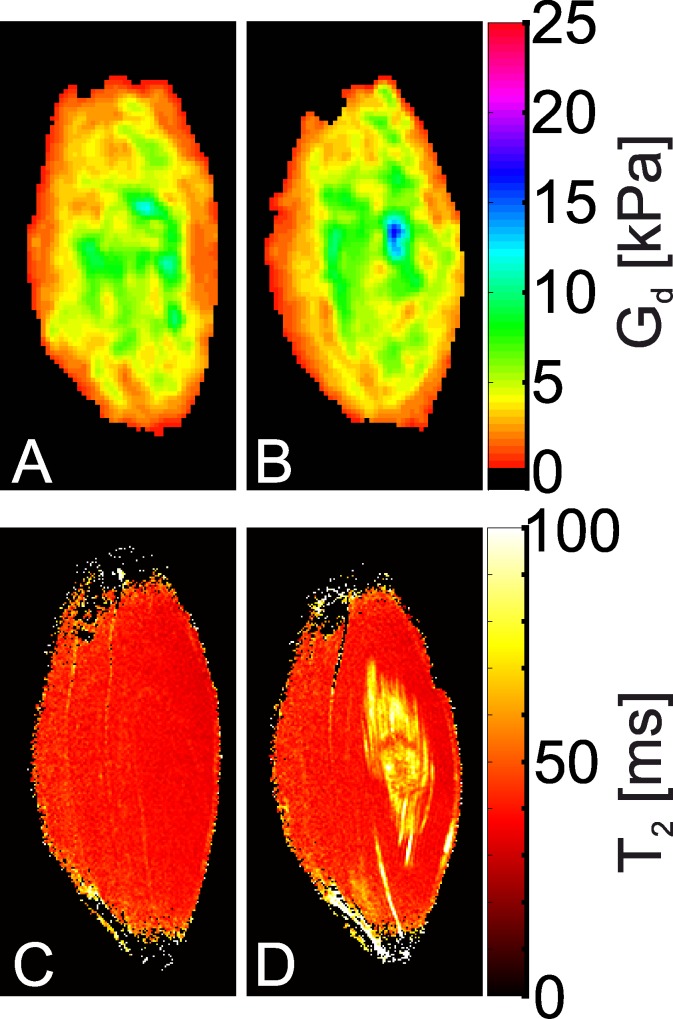
Elastograms and T_2_ maps before and after indentation. (**A, B**) Elastograms representing shear storage modulus G_d_ and (**C, D**) T_2_-maps (**A, C**) before, and (**B, D**) after indentation.

In Fig 8, representative MRE elastograms (Fig 8A) before, as well as (Fig 8B) after 2 h of indentation are shown together with the corresponding T_2_-maps (Fig 8C and 8D). Increased shear storage modulus G_d_ was observed in the TA in the elastogram that was measured following 2 h of indentation. T_2_-maps after indentation revealed a large region of elevated T_2_ values in the TA muscle, indicative for edema and muscle injury. The region of increased shear storage modulus G_d_ was less diffuse and smaller compared to the region of elevated T_2_. The region of high shear storage modulus G_d_ highlights a central focus with severe muscle damage, whereas T_2_ elevation occurs in a larger region with edema surrounding the injury. The MRE elastograms of the other analyzed animals show the same consistent results.

**Fig 9:**
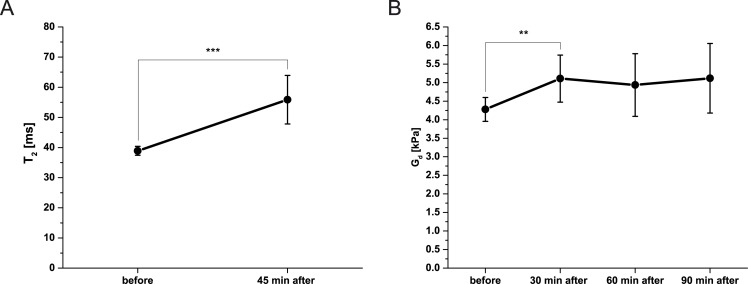
Mean (± sd) T_2_ and G_d_ measured in the circular ROI of 4x indentor’s diameter positioned around the center of indentation of 6 animals before and after end of indentation. (**A**) Mean (± sd) T_2_ before and 45 min after end of indentation. T_2_ 45 min after end of indentation was significantly higher (***p < 0.01) than before indentation. (**B**) Mean (± sd) G_d_ before, 30, 60 and 90 min after end of indentation. G_d_ is increased at all time points after indentation. G_d_ at 30 min after end of indentation was significantly higher than before indentation (**p < 0.05).

**Fig 10:**
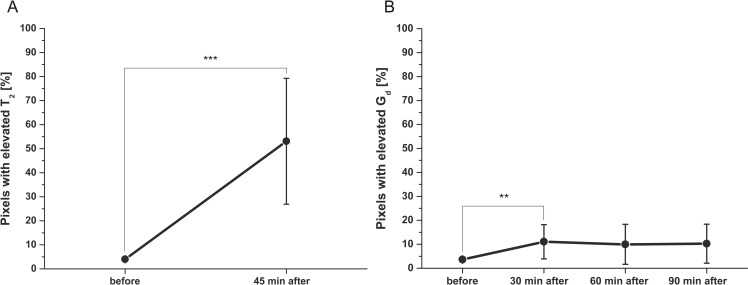
Percentage elevated T_2_ and G_d_ pixels of ROI in the TA muscle of 6 animals before and after end of indentation. **Pixels were selected as significantly elevated, if the value was higher as mean value before indentation + 2 x sd.** (**A**) Percentage elevated T_2_ pixels before and 45 min after end of indentation. Percentage elevated T_2_ pixels 45 min after end of indentation was significant higher (***p < 0.01) than before indentation. (**B**) Percentage elevated G_d_ pixels before, 30, 60 and 90 min after end of indentation. Percentage elevated G_d_ pixels 30 min after end of indentation was significant higher than before indentation (**p < 0.05).

For all 6 animals measured before and after end of indentation, the shear storage modulus G_d_ maps of the TA muscle were reconstructed at four time points: before, 30 min after, 60 min after, and 90 min after end of indentation. Two animals had missing data at the 90 min after time point. The T_2_ maps were calculated at two time points: before and 45 min after end of indentation. T_2_ and G_d_ cannot be measured at the same time, particularly because of the long, 17 min, G_d_ acquisition. As a result fewer time points were chosen for T_2_ than G_d_ as the latter is of more interest in this study. G_d_ and T_2_ values in the circular ROI around the indentor are shown in Fig 9. The percentage of significant elevated G_d_ and T_2_ pixels measured in the whole TA ROI are displayed in Fig 10. Measurements of mean (± sd) and percentage elevated pixels of G_d_ and T_2_ are also summarized for each animal in Tables 2 and 3. G_d_ Standard deviation (0.1 kPa) and coefficient of variation (3%) were low at baseline in non-damaged TA muscle.

**Table 2:** Measured mean (±sd) shear storage modulus G_d_, and percentage elevated pixels of shear storage modulus G_d_ elev. in ROI of whole TA muscle, and mean shear storage modulus G_d_ in circular ROI of 4x indentor’s diameter.

animal	before	30 min after			60 min after			90 min after		
	Whole TA	Whole TA		Ind.	Whole TA		Ind.	Whole TA		Ind.
	G_d_	G_d_	G_d_elev.	G_d_	Gd	G_d_elev.	G_d_	G_d_	G_d_elev.	G_d_
	[kPa]	[kPa]	[%]	[kPa]	[kPa]	[%]	[kPa]	[kPa]	[%]	[kPa]
**1**	4.4 (1.4)	4.5 (1.8)	8.2	4.4	4.5 (1.6)	7.3	4.3			
**2**	4.2 (1.6)	4.6 (1.6)	7.4	4.7	4.4 (1.7)	6.0	4.5			
**3**	4.1 (1.8)	4.3 (1.9)	7.8	4.9	3.9 (1.6)	2.3	4.3	4.0 (1.5)	2.5	4.4
**4**	4.2 (1.7)	4.8 (2.8)	12.2	5.3	4.4 (2.1)	6.8	4.8	4.4 (2.1)	8.2	4.5
**5**	4.1 (1.8)	5.8 (2.9)	25.0	6.2	5.9 (3.0)	26.0	6.5	5.7 (3.0)	21.8	6.4
**6**	4.1 (2.1)	5.1 (2.1)	5.8	5.4	5.0 (2.3)	11.5	5.3	4.8 (2.3)	8.6	5.2
**mean**	4.2	4.8	11.1**	5.1**	4.7	10.0	4.9	4.7	10.3	5.1
**sd**	0.1	0.6	7.2	0.6	0.7	8.4	0.9	0.7	8.2	0.9
**CV**	3	12	65	13	15	84	12	15	80	18

G_d_ elev., percentage of pixels with elevated G_d_ (> mean G_d_ before + 2 x sd); Ind.,circular ROI of 4x indentor’s diameter

** p < 0.05 with paired sampled t-test.

**Table 3:** Measured mean (±sd) and percentage elevated pixels of T_2_ map in ROI of whole TA muscle, and mean T_2_ in circular ROI of 4x indentor’s diameter.

animal	before	45 min after		
	Whole TA	Whole TA		Ind.
	T_2_	T_2_	T_2_elev.	T_2_
	[ms]	[ms]	[%]	[ms]
**1**	43 (6)	47 (11)	15.2	49
**2**	38 (4)	65 (20)	79.4	69
**3**	38 (5)	57 (23)	65.7	57
**4**	39 (5)	54 (22)	47.4	52
**5**	40 (5)	69 (36)	78.8	60
**6**	39 (4)	47 (14)	32.0	49
**mean**	39	57***	53.1***	56***
**sd**	2	9	26.2	8
**CV**	4	16	49	14

T_2_ elev., percentage of pixels with elevated T_2_ (> mean T_2_ before + 2 x sd); Ind.,circular ROI of 4x indentor’s diameter

*** p < 0.01 with paired sampled t-test.

Averaged across all animals, the mean T_2_ of the TA muscle at 45 min after end of indentation was significantly higher than before indentation (p < 0.01). A trend of increased mean shear storage modulus G_d_ after indentation was apparent. Mean shear modulus G_d_ at 30 min after indentation was significantly higher than before indentation (p < 0.05).

The percentage significantly elevated T_2_ and G_d_ pixels after end of indentation was significantly higher compared to before indentation (T_2_; p < 0.01, G_d_; p < 0.05 at 30 min after). The percentage elevated T_2_ pixels after indentation was significantly higher compared to the percentage elevated G_d_ pixels after indentation at all three time points (p < 0.01, p < 0.01, p < 0.05). In both T_2_ and G_d_ maps before indentation a comparable percentage of significantly elevated pixels was found (3.6 ± 0.6% for G_d_ and 4.0 ± 0.7% for T_2_).

## Discussion

In this paper we have presented a combined Magnetic Resonance (MR) compatible indentation setup and MR Elastography (MRE) setup to study the spatial-temporal changes in mechanical properties that result from deformation-induced injury. The setup was designed in such way that tissue indentation and the MRE methods can either be used separately or simultaneously. The setup was successfully tested in a deep tissue injury rat model by damage-inducing indentation and MR Elastography measurements of the rat TA muscle. Results show that it is possible to quantitatively measure muscle mechanical properties over time. The elastograms after 2 h of indentation revealed a local increase in shear storage modulus G_d_ after indentation. The spatial extent of change in stiffness was also evaluated by quantifying the percentage pixels with significantly enhanced G_d_ values with respect to baseline. Quantification of G_d_ values in a circular ROI around the indentation showed a significant elevation of G_d_ values 30 min after indentation. At 60 and 90 min after indentation a trend of elevated G_d_ was observed. The determined mean shear moduli values in non-damaged condition are in range with reported values of MRE measurements and *ex vivo* biomechanical tests [23,71,87–89]. These results show the high potential of the setup to measure non-invasively the quantitative mechanical properties related to deep tissue injury *in vivo*.

There are still options to further improve the indentation setup. Firstly, the setup does not allow for an indentation controlled from outside the scanner; i.e. currently the setup has to be taken out of the scanner bore to apply, adjust or remove the indentor. This manual operation, especially if multiple indentation depths are of interest, is time consuming and repositioning of the setup following indentor adjustment may cause misalignment of image data sets. Image registration can be employed to correct for such misalignment. However, a controlled indentation device would be preferred. Also, the indentor has no force sensor incorporated in the current implementation. Although indentation control and force sensing can be achieved inside an MRI scanner (e.g. see [90] for a clinical scanner set-up) these were not implemented in the current study due to the space challenges in a small animal MR scanner. Knowledge of the indentation force boundary conditions, combined with MRI data of the undeformed and deformed configuration would enable inverse FEA based investigation of hyperelastic tissue mechanical properties [91].

The MRE part was designed to be flexible in feet-head and anterior-posterior direction, but is limited in left-right direction due to the fixed position of the electromagnetic shaker driving the MRE part. In the positioning of the leg of the animal this limitation was taken into account, which resulted in successful coupling of the MRE actuator piston in all animals. Although only MRE measurements of 900 Hz were presented here, higher and lower frequencies (100 Hz– 1500 Hz) were also evaluated. Frequencies between 1000 and 1500 Hz did not always result in sufficient displacement (< 0.5 μm displacement) to produce waves through the whole TA muscle. At 900 Hz a decaying wave with displacement in the range of 0.5–10 μm (mean 2 μm) was induced. This displacement amplitude is comparable to other MRE studies [87,92]. We observed that the MRE actuation system behaves frequency dependent; less power was needed with lower frequencies to generate sufficient displacement at the MRE piston. In addition, tests with a prototype of the current MRE actuator showed eigenfrequencies with different designs of cantilevers resulting in higher MRE piston amplitudes [80]. Altogether, measurements at each frequency in the range 100 Hz– 1500 Hz are possible but further optimization to obtain the correct amplitude at the MRE piston is required.

The alginate cast allowed proper fixation of the animal leg with anatomical perfect fit, provided boundary condition information for FEA and provided good susceptibility matching. The use of alginate has advantages over plaster [93–95]. Alginate does not clot, is easy to prepare, can be molded anatomically around the rat leg and is very easy to remove without any remnants. In addition, the surrounding alginate provided appropriate susceptibility matching beneficial for MRI, i.e. it reduced artefacts and improved image quality. For this reason, the MRE measurements during indentation, where the top of the alginate cast was removed, was performed with SE-MRE instead of SE-EPI-MRE. SE-MRI is less sensitive to field inhomogeneities but more time consuming. A typical SE-MRE scan with three encoding directions plus reference will take approximately 1h. To capture dynamic changes in tissue mechanical properties SE-EPI-MRI is therefore preferred.

After application of the indentor to the TA muscle inside the MRI scanner, increased signal intensity in the TA muscle was observed on T_2_ maps as a result of edema, resulting from the induced damage and associated inflammatory response [22–26]. ROI analysis showed a significant increase in T_2_ after end of indentation. A significant increase in the percentage of significantly elevated T_2_ pixels was observed after indentation. The area of pixels with elevated T_2_ at 45 min after indentation was significantly larger than those with elevated G_d_ at 30, 60 and 90 min after indentation. Using T_2_ alone as specific damage area indicator might result in overestimation of the size of the damaged area, as in the initial injury phase fluids might diffuse between the fibers proximally and distally from the wound. The size of the affected region in T_2_ in comparison to G_d_ may provide insights in the causes and consequences of deformation induced muscle damage.

In the current study deep tissue injury and skeletal muscle tissue were of interest. Shear moduli data for the more superficial adipose tissue were not derivable in the current study because fat suppression techniques were employed for the MR acquisitions, enabling minimization of chemical shift artefacts. If such data is however desired non-EPI MRE sequences can be employed without the use of fat suppression.

The employed MRE inversion algorithm assumes Hooke’s law of linear (visco) elasticity (as is valid for the small strain vibrational motions) and assumes isotropic tissue behavior. However, as is the case for muscle tissue, the epi-, peri- and endomysium structure causes the tissue to be anisotropic. Furthermore, damage and deformation may independently cause changes in elastic symmetry and therefore induce anisotropy. Since the inversion algorithm assumes isotropy the reconstructed shear moduli information may potentially be affected by any underlying anisotropy. Exactly how anisotropy is reflected in analyses based on isotropic inversion methods is currently unknown, and may also relate to the directions wave happen to locally follow. However, when combined with MRI-compatible force sensing, diffusion tensor-based fiber architecture assessment, and inverse FEA, the presented methods do offer a means to assess such effects in the future [86,90,96–98]. This is because the initial anisotropy and non-linear elastic behavior would be accurately reflected in FEA based on such measurements, and elastic tensor data for any state of deformation can be directly compared to MRE findings. In principle such analysis can be coupled with FEA incorporating constitutive formulations of damage and swelling.

In conclusion, we have presented an MRI-compatible setup for studying the mechanical properties of rat TA muscle. The setup allows for controlled deformation under MRI-guidance, monitoring of the wound development by MRI, and quantification of tissue mechanical properties by MRE. We expect that improved knowledge of changes in soft tissue mechanical properties due to deep tissue injury, will provide new insights in the etiology of deep tissue injuries, skeletal muscle damage and other related muscle pathologies. Future studies will focus on the pathophysiological processes behind the changes in mechanical properties of damaged skeletal muscle and evaluation of the changing mechanical properties over time.

## Ethical approval

All animal experiments were approved by the Animal Care and Use Committee of Maastricht University, Maastricht, The Netherlands (protocol 2013–047, Maastricht University, Maastricht, The Netherlands) and performed in accordance with the Directive 2010/63/EU for animal experiments of the European Union.

## Supporting Information

S1 Fig Photograph of a rat positioned in the setup. Rat, Indentation and MRE component are indicated with arrows. In **A**, the indentor, put through the surface RF coil, positioned on top of TA muscle in rats hindleg, and the MRE piston attached at distal side of TA muscle are shown. Indentation component is removed in **B**, revealing the MRE component and surface coil. Pre-amplifier block of the surface coil, the respiratory sensor and the rectal temperature probe are also visible. Anesthesia mask and rat’s head are underneath the pre-amplifier block.
(TIF)

S2 Fig Detail of indentation and MRE actuator part of setup. Following parts are labeled: u-shaped profile (**a**), cutout for the rat’s groin (**b**), indentor (**c**), movable indentor holder (**d**), rotatable half arch (**e**), dovetail profile (**f**), spacer plates (**g**), MRE piston (**h**), drive rod (**i**), cantilever (**j**).
(TIF)

S3 Fig MRE pre and post indentation movie. Movie of a 16 offsets 900 Hz SE-EPI-MRE (**A**) before and (**B**) after indentation.
(AVI)
