# Supplementary figures and images for: A MRI-Compatible Combined Mechanical Loading and MR Elastography Setup to Study Deformation-Induced Skeletal Muscle Damage in Rats
Source: PLoS One. 2017 Jan 11;12(1):e0169864. doi: 10.1371/journal.pone.0169864 (PMC5226723; doi:10.1371/journal.pone.0169864)

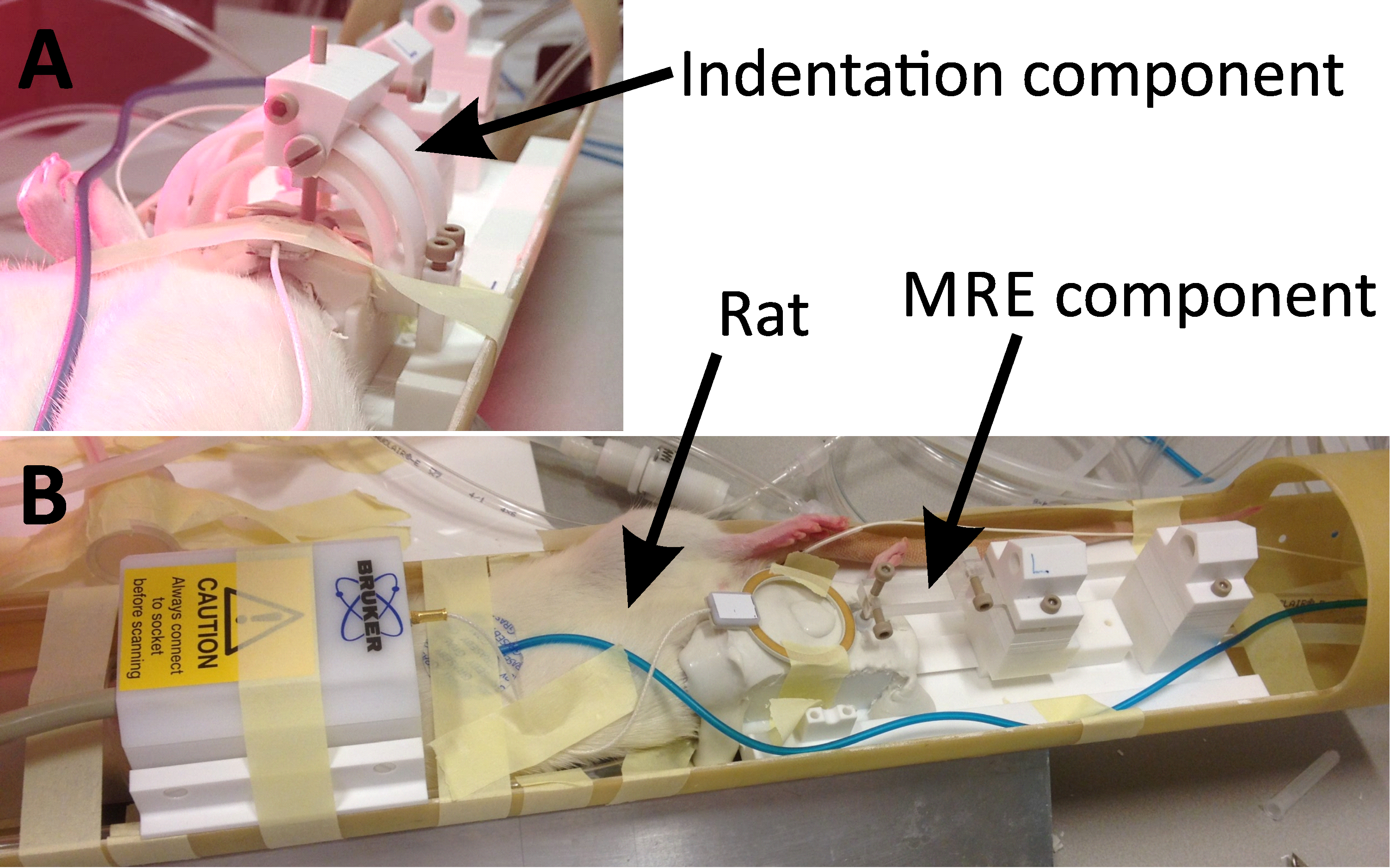

Supplement: S1 Fig — Rat, Indentation and MRE component are indicated with arrows. In A, the indentor, put through the surface RF coil, positioned on top of TA muscle in rats hindleg, and the MRE piston attached at distal side of TA muscle are shown. Indentation component is removed in B, revealing the MRE component and surface coil. Pre-amplifier block of the surface coil, the respiratory sensor and the rectal temperature probe are also visible. Anesthesia mask and rat’s head are underneath the pre-amplifier block. (TIF) [file pone.0169864.s001.tif]

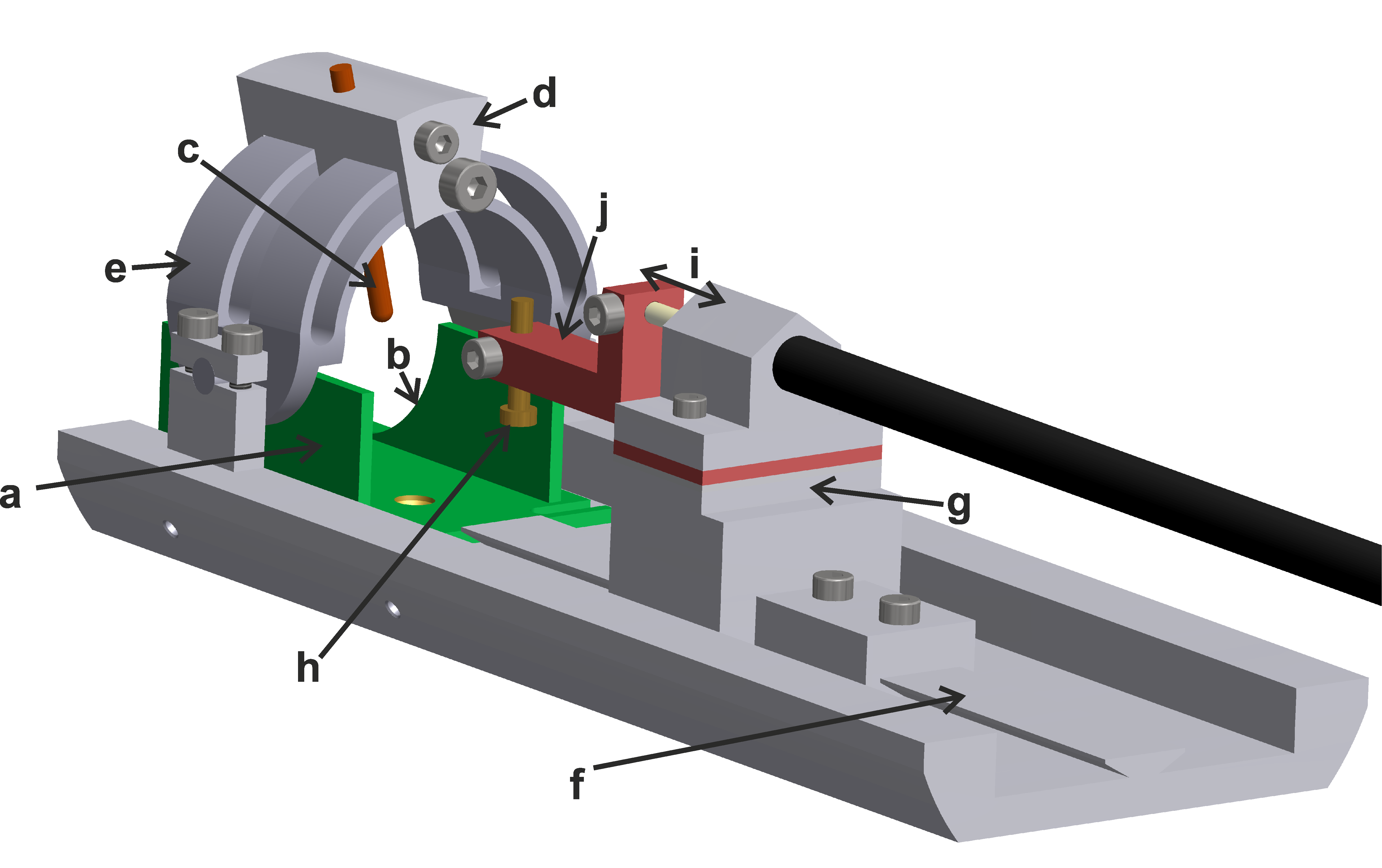

Supplement: S2 Fig — Following parts are labeled: u-shaped profile (a), cutout for the rat’s groin (b), indentor (c), movable indentor holder (d), rotatable half arch (e), dovetail profile (f), spacer plates (g), MRE piston (h), drive rod (i), cantilever (j). (TIF) [file pone.0169864.s002.tif]
